# Calcineurin and Protein kinase G regulate *C. elegans *behavioral quiescence during locomotion in liquid

**DOI:** 10.1186/1471-2156-11-7

**Published:** 2010-01-27

**Authors:** Rajarshi Ghosh, Scott W Emmons

**Affiliations:** 1 Lewis Sigler Institute of Integrative Genomics, 110 Carl Icahn Laboratory, Princeton University, Princeton, NJ 08544, USA; 2 Department of Genetics, Albert Einstein College of Medicine, Bronx, New York 10461, USA

## Abstract

**Background:**

Most rhythmic motor behaviors in nature are episodic i.e. they alternate between different behavioral states, including quiescence. Electrophysiological studies in invertebrate behavioral switching, maintenance and quiescence have elucidated several neuronal mechanisms that generate a temporal pattern in behavior. However, the genetic bases of these processes are less well studied. We have previously uncovered a novel episodic behavior exhibited by *C. elegans *in liquid media where they alternate between distinct phases of rhythmic swimming and quiescence. Here, we have investigated the effect of several genes and their site of action on the behavioral quiescence exhibited in liquid by the nematode *C. elegans*.

**Results:**

We have previously reported that high cholinergic signaling promotes quiescence and command interneurons are critical for timing the quiescence bout durations. We have found that in addition to command interneurons, sensory neurons are also critical for quiescence. We show that the protein phosphatase calcineurin homolog *tax-6 *promotes swimming whereas the protein kinase G homolog *egl-4 *promotes quiescence. *tax-6 *expression in the sensory neurons is sufficient to account for its effect. *egl-4 *also acts in multiple sensory neurons to mediate its effect on quiescence. In addition our data is consistent with regulation of quiescence by *egl-4 *acting functionally downstream of release of acetylcholine (ACh) by motor neurons.

**Conclusions:**

Our study provides genetic evidence for mechanisms underlying the maintenance of a behavioral state operating at multiple neuronal levels through the activities of a kinase and a phosphatase. These results in a genetically tractable organism establish a framework for further dissection of the mechanism of quiescence during episodic behaviors.

## Background

Most natural behaviors are characterized by transitions between distinct behavioral states determined by the internal state of an organism as well as spatial and temporal variance of the environment. Observations of leech long-term behavior in an unstructured environment have resulted in identification of several behavioral states, including stationary states, and transitions between these states that depend only on the most recent prior behavioral state [1,2]. Invertebrate electrophysiological studies of a given behavioral state such as quiescence and switching between behavioral states *e.g*. swimming and crawling in *Tritonia *[3,4], swallowing and quiescent states in *Lymnea *[5] among many others have elucidated several neuronal mechanisms. However the genetic basis of these processes are less well studied.

Despite a single gait underlying locomotion in liquid and solid media [6], referred to as swimming [7,8] and crawling respectively, nematodes exhibit distinct behavioral patterns when observed for long periods of time in liquid [9,10]. We have previously described that in liquid, wild type worms display periods of active swimming alternating with a quiescent state lasting for several minutes [9]. We previously showed that quiescence in liquid results from high cholinergic signaling downstream of motor neurons and that the maintenance of the quiescent state requires command inter-neurons [9].

In addition to the quiescent state exhibited by worms in liquid [9], two other behavioral quiescent states have been studied in *C. elegans *[11-13]. Movement of *C. elegans *stops when they undergo lethargus, a developmental state that occurs before each of the four molts during its life cycle [11,12]. This quiescent state exhibits characteristics that resemble sleep in other organisms [11]. On agar plates with high-quality food, movement and feeding of an adult worm is interjected by stops, a behavioral characteristic that has parallels to satiety in mammals [13]. Molecularly, two different signaling systems have been identified to influence these quiescent states. Insulin/TGF-β and EGF signaling induce quiescence on plates with high quality food and during lethargus respectively [11-13]. In both quiescence paradigms, protein kinase G (PKG) activity in sensory neurons is required to promote quiescence. Eventually the activity of PKG at the sensory neuronal level must modulate activities of the motor and/or interneurons to bring about quiescence. How PKG activity in sensory neurons drives quiescence at the motor/interneuron levels is not known. Also it remains to be seen how much overlap exists in the genetics of the three behavioral quiescence states that have been described. The unique features of long term swimming behavior, ease of genetic analysis and the knowledge of the pattern of synaptic connectivity of the entire nervous system [14] that allow mapping of molecular to neuronal correlates underlying behavior makes *C. elegans *a suitable model to dissect the molecular mechanisms underlying this long-term behavioral pattern.

To further investigate the underlying mechanism(s) of quiescence in liquid, we examined the cellular site of action of several genes that had important effects on swim/quiescence cycling. Several of these do not act at the motor neuron-muscle circuitry but instead act in sensory neurons. We found that maintenance of swimming requires calcineurin activity in the sensory neurons. The PKG homolog *egl-4 *promotes quiescence by acting at two levels: in the sensory neurons and also downstream of command interneurons. Our analysis suggests that there may be two mechanisms by which swimming motions may be stopped, one by blocking output of the motor system at the level of motor neurons and muscles, a mechanism that appears to come into play when worms swim in liquid, and one by inhibition of initiation of swimming motions upstream of this block, which is strongly influenced by sensory input.

## Results

### PKG is required for maintenance of the quiescent state during swimming in liquid

**Figure 1:**
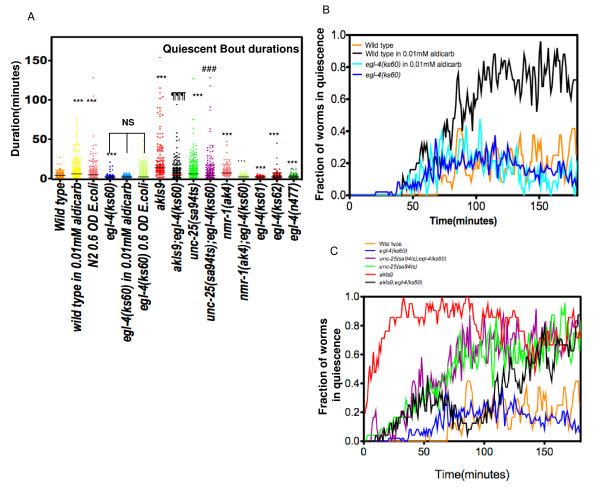
**PKG promotes quiescence**. A) Scatterplot of quiescent bouts for wild type, wild type in presence of 0.01 mM aldicarb (n = 23), wild type in 0.6 O.D *E.coli *Op50 (n = 30), *egl-4(ks60) *(n = 24), *egl- 4(ks60)*in 0.01 mM aldicarb (n = 23), *egl-4(ks60) *in 0.6 OD Op50 (n = 24), *nmr-1(ak4) *(n = 33), *egl-4(ks60);nmr-1(ak4) *(n = 24), *egl-4(ks61) *(n = 24), *egl-4(ks62) *(n = 46) and *egl-4(n477) *(n = 22). The medians are shown by a black or a red horizontal line (for *akIs9*). *** significantly different from wild type, "' significantly different from *nmr-1(ak4)*, ### significantly different from *unc-25(sa94ts) *¶¶¶ significantly different from *unc-25(sa94ts)*. Mann-Whitney U test for each pair. NS nonsignificant difference at p < 0.001. B) Fraction of worms in quiescence versus time for wild type (n = 25), wild type in presence of 0.01 mM aldicarb (n = 23), *egl-4(ks60) *(n = 24), *egl-4(ks60)*in 0.01 mM aldicarb (n = 23). C) Fraction of worms in quiescence versus time forwild type (same as Figure 1B), *unc-25(sa94) *(n = 24), *egl-4(ks60) *(same as Figure 1B), *egl-4(ks60);unc- 25(sa94)*(n = 24), *akIs9*(n = 21) and *akIs9;egl-4(ks60)*(n = 27).

The PKG homolog of *C. elegans*, *egl-4*, regulates long-term olfactory adaptation [15], different locomotory states (roaming and dwelling) [16] and promotes longer duration of quiescence bouts under different paradigms [11-13]. Though it is widely expressed in the neuro-muscular system and has been implicated in modulation of acetylcholine release at neuromuscular junctions [17], the pleiotropic effects of *egl-4 *have been mostly attributed to its activity in sensory neurons [15-18]. We have previously shown that loss-of-function alleles of *egl-4 *affected maintenance of the naturally occurring quiescent bouts that occur with regular periodicity when a worm is swimming in liquid [9]. Indeed, when compared to the wild type animals, the median fraction of *egl- 4(ks60) *animals that remained in quiescence during the second phase of swimming (phase one is defined as the first ninety minutes from the start of the assay and the subsequent 90 minutes is defined as the second phase of the assay) was significantly lower (wild type: 0.21; *egl-4(ks60)*: 0.14, p < 0.0001, Mann Whitney test, trends are shown in Figure 1B). Here we show that the loss-of-function mutant, *egl-4(ks60)*, suppresses increased quiescence resulting from including 0.01 mM aldicarb in the assay medium (Figure 1A, B). The median quiescent bout durations of *egl-4(ks60) *in 0.01 mM aldicarb did not differ from *egl-4(ks60) *in buffer alone (Figure 1A, Mann Whitney U test, p = 0.5683). This suppression was absent at higher doses (0.1 or 1 mM) aldicarb (data not shown). However *egl-4 *mutants still went into quiescence albeit for a shorter duration in 0.01 mM aldicarb. Taken together these data suggest that increased quiescent bout durations in liquid due to increased cholinergic activity require *egl-4 *function.

The increased cholinergic activity during quiescence appeared by several criteria to be at the neuromuscular junction [9]. During quiescence in liquid body-wall muscles are uniformly contracted. Quiescence induced by aldicarb was enhanced by mutation in *dgk-1*, which increases ACh release from motor-neurons [9,19]. Mutations resulting in reduced synthesis/release of GABA by GABAergic motor neurons, which opposes the action of ACh on muscles, also increased quiescence, whereas the GABA agonist muscimol promoted swimming. Since *egl-4 *is necessary for ACh- mediated quiescence, we conclude that *egl-4 *has an activity that promotes quiescence functionally downstream of ACh release by motor neurons.

### Further evidence for PKG activity downstream of motor neurons in regulating quiescence

In order to further investigate the quiescence mechanism that appeared to require *egl-4 *function downstream of ACh release by motor neurons, we examined the relationship of *egl-4 *function and additional components of the motor circuitry. We had previously found that mutations resulting in defects in GABAergic signaling result in increased fraction of worms in quiescence [9]. To determine whether quiescence induced by decreased GABA required *egl-4*, we examined double mutants with the GABA-deficient mutant *unc-25(sa94ts) *[20]. Unlike its effect on quiescence induced by Ach, *egl-4(ks60) *had little effect on increased quiescence due to loss of GABA activity. Although the median quiescent bout duration of *unc-25(sa94) *was (6, mean = 9.6 ± 0.7 minutes) that was slightly longer than the median quiescent bout duration of *unc-25(sa94); egl-4(ks60) *(4.0, mean = 7.5 ± 0.7 minutes) (p < 0.0001, Mann-Whitney U test), the distribution of the quiescent bout durations for *unc-25(sa94) *and *unc-25(sa94); egl-4(ks60) *exhibited considerable overlap (Additional File 1). Worms of both these genotypes exhibited long quiescent bouts from which they did not initiate swimming reflected by their overlapping trends of fraction of worms in quiescence and distribution of quiescent bouts (Figure 1C and Additional File 1). We conclude that loss of function of *egl-4 *was not sufficient to suppress the increased quiescence due to *unc-25(sa94) *mutation (Table 1) indicating that *egl-4 *primarily acts upstream of GABA signaling in regulating duration of quiescence.

**Table 1:** Quiescent Bout Durations

STRAIN	Mean (min)	Median (min)	SD	SE	Number of bouts
*Wild type*	4.8	4.0	2.2	0.1	489
*cnb-1(jh103)*	20.5	1.0	49.0	3.5	202
*tax-6(db60)*	5.5	1.0	13.3	0.7	214
*tax-6(db60)(tax-6cDNA, muscle+)*	10.1	1.0	25.8	1.6	261
*tax-6(db60)(tax-6 sensory+inter+)*	4.7	4.0	4.4	0.3	218
*tax-6(db60)(tax-6 sensory+inter-)*	3.9	3.0	3.3	0.2	192
*egl-4(ks60)*	2.7	2.0	2.5	0.2	173
*egl-4(ks60) in 0.6 OD E.coli(OP50)*	4.6	2.0	5.8	0.3	461
*Wild type in 0.6 OD E.coli(OP50)*	9.3	5.0	15.7	1.1	189
*akIs9*	25.9	14.5	29.8	2.4	158
					
*akIs9;egl-4(ks60)*	8.9	5.0	11.5	0.8	206
*nmr-1(ak4)*	9.0	7.0	6.1	0.5	170
*unc-25(sa94)*	9.6	6.0	11.7	0.7	309
*unc-25(sa94);egl-4(ks60)*	7.5	4.0	13.5	0.7	352
*nmr-1(ak4);egl-4(ks60)*	2.8	2.0	1.6	0.1	222
*osm-6(p811)*	7.0	5.0	7.4	0.3	479
*osm-3(p802)*	7.4	6.0	6.2	0.5	165
*osm-6(p811);egl-4(ks60)*	3.8	3.0	2.9	0.1	452
*osm-3(p802);egl-4(ks60)*	2.9	3.0	1.8	0.1	691
*nmr-1(ak4);osm-6(p811)*	14.8	9.0	16.3	1.1	221
*tax-2(p691);tax-4(ky89)*	4.2	4.0	1.9	0.1	281
*egl-4(n479)*	2.1	1.0	1.5	0.3	30
*egl-4(n479) [odr-3p::egl-4] NQ2*	3.0	3.0	2.3	0.5	19
*egl-4(n479) [odr-3p::egl-4] NQ5*	4.4	3.0	4.5	0.7	48
*egl-4(n479) [odr-3p::egl-4] NQ7*	2.9	2.0	2.1	0.7	9
*egl-4(n479) [odr-1p::egl-4] NQ11*	3.7	1.0	4.2	0.6	47
*egl-4(n479) [odr-1p::egl-4] NQ13*	3.6	2.0	3.6	0.5	55
*egl-4(n479) [tax-4p::egl-4] NQ20*	6.6	2.0	15.1	1.6	91
*egl-4(n479) [tax-4p::egl-4] NQ21*	7.4	6.0	6.1	1.2	24
*egl-4(n479) [tax-4p::egl-4] NQ22*	4.5	2.0	6.5	0.6	114

Summary statistics of quiescent bout durations of strains used in the study The mean, median, standard deviation and the standard error of the mean for the quiescent bout durations in the first 180 minutes of the assay are shown. 'min' refers to minute.

We previously found that command interneurons regulate the timing of quiescence bouts [9]. Consistent with a role of *egl-4 *downstream of command interneurons, increased quiescence in transgenic strains expressing a leaky glutamate channel in the command neurons plus 12 classes of additional neurons (*akIs9*) causing constitutive depolarization of command interneurons [21], were partially suppressed by *egl-4(ks60) *(Figure 1A, C). Taken together, these observations suggest a role of *egl-4 *genetically downstream of command interneurons in sustaining quiescence. We also observed that suppression of the increased quiescence due to *akIs9 *by *egl-4(ks60) *was more potent during the first ~90 minutes of the assay (Figure 1C). Thus *egl-4 *independent mechanisms are required to shape the behavioral pattern beyond that time period. Overall, these results place a site of action for activity of *egl-4 *required for quiescence during swimming downstream of command interneurons and release of ACh by motor neurons, but upstream of GABA signaling.

Consistent with a role of command interneurons in timing quiescent bout duration, we found that a deletion in *nmr-1*, encoding NMDA receptor subunit expressed in the ring motor neuron RIM and all command interneurons except AVB [22], resulted in dramatically increased durations of quiescent bout (Figure 1A). The median quiescent bout durations of *nmr-1(ak4)*; *egl-4(ks60) *were similar to *egl-4(ks60) *indicating *egl-4 *acts downstream of or within command interneurons in maintenance of quiescence (Figure 1A).

### PKG acts in sensory neurons to modulate the quiescent state

**Figure 2:**
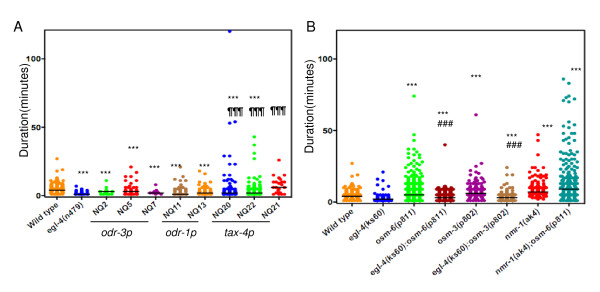
***egl-4 *acts in sensory neurons to promote quiescence**. A) Scatterplot of quiescent bouts for wild type (same as Figure 1A), *egl-4(n479) *and *egl-4 *rescue strains. The strains starting with NQ are transgenic strains expressing *egl- 4 *cDNA under *odr-3 *(NQ2, NQ5 and NQ7), *odr-1*(NQ11 and NQ13) and *tax-4 *(NQ20, NQ21 and NQ22) promoters. *egl-4(n479) *(n = 12), NQ2 (n = 13), NQ5 (n = 17), NQ7 (n = 12), NQ11 (n = 16), NQ13 (n = 16), NQ20 (n = 18), NQ21(n = 10) and NQ22 (n = 24). *** significantly different from wild type. ¶¶¶ significantly different from *egl- 4(n479) *Kruskal-Wallis test followed by Dunn's multiple comparison. B) Distribution of quiescent bouts for wild type (as in Figure 1A), *egl-4(ks60) *(same as Figure 1A), *osm-6(p811) *(n = 33), *egl-4(ks60);osm-6(p811) *(n = 47), *osm- 3(p802)*(n = 31) and *egl-4(ks60);osm-3(p802)*(n = 30), *nmr-1(ak4) *(same as Figure 1A) and *nmr-1(ak4);osm-6(p811) *(n = 34), *** and ### p < 0.0001 by Mann-Whitney U test, significantly different from wild type and *egl-4(ks60) *respectively.

To investigate whether *egl-4 *has an activity in the sensory neurons in regulating quiescence, we expressed an *egl-4 *transgene under different promoters in subsets of sensory neurons in *egl-4(n479) *loss-of-function mutant background. Like other *egl-4 *lossof- function mutants (Figure 1A), these worms had significantly reduced duration of quiescence bouts compared to the wild type worms (Figure 2A, Wilcoxon signed rank test, p < 0.0001). When an *egl-4 *transgene was expressed under a *tax-4 *promoter there was a partial rescue of this mutant phenotype, the median quiescence bout did not differ from the wild type strain N2 (2 out of 3 transgenic lines) (Figure 2A). However, in all three lines there were long quiescent bouts (>8 minutes, NQ20: 17.6%, NQ21: 41.7%, NQ22: 9.7%) that were not observed in the wild type (3.4% of all bouts), or in *egl-4(lf) *mutants (0%). In contrast, no rescue was obtained by expression from *odr-1 *and *odr-3 *promoters (Figure 2A). Further, long quiescent bouts did not occur or were only somewhat increased under the odr-3 (NQ2: 5.2%, NQ5:14.5%, NQ7: 0%) and odr-1 (NQ11:10.6%, NQ13: 9.1%) promoters. All three promoters drive the expression of the *egl-4 *transgene in twosensory neurons AWB and AWC, while the *tax-4 *and *odr-3 *promoters drive expression in 8 and 3 additional pairs of sensory neurons respectively [11,15,16,23]. Thus *tax-4 *expressing neurons define a site of action of *egl-4 *in sustaining quiescent bouts.

Consistent with a role of sensory neurons in quiescence, we found that quiescence duration could be increased by defects in sensory physiology due to mutations in *osm-6 *or *osm-3 *(critical for proper cilium structure in sensory neurons). Both of these mutations result in abnormal sensory neuron structure [24] (Figure 2B, Table 1). Increased quiescence in *osm-3 *and *osm-6 *mutants was suppressed by *egl-4(ks60) *mutation (Figure 2B) indicating that *egl-4 *acts downstream or in sensory neurons. In addition we previously reported that inclusion of food (0.6 O.D. *E.coli(OP50)*) increased the quiescent bout durations [9]. The increased quiescence bout duration of wild type worms induced by inclusion of *E.coli *in the assay media was also significantly reduced in *egl-4(ks60) *mutants (Figure 1A, Table 1).

Since both sensory neurons and command interneurons contribute towards sustaining quiescence and thus there appeared to be two sites of action of *egl-4*, we asked whether these two pathways were independent. We assayed double mutants of *nmr- 1(ak4);osm-6(p811)*. The quiescence bouts in the *nmr-1(ak4);osm-6(p811) *mutants were significantly longer than either of the single mutants alone (Figure 2B, Table 1). This additive role of *nmr-1 *and *osm-6 *is consistent with two independent pathways of quiescence maintenance.

### Calcineurin activity in sensory neurons is essential for swimming in liquid

*tax-6 *and *cnb-1*, the *C. elegans *homologues of subunits of calcineurin phosphatase subunits A and B respectively, are expressed in many tissues, including sensory neurons, interneurons and muscles [25,26]. *C. elegans *calcineurin has been implicated in diverse behaviors including locomotion, thermosensation and chemosensation [25,26]. Calcineurin negatively affects several sensory behaviors. For example, loss of *tax-6 *results in hyper-activation of sensory neurons in an olfactory adaptation paradigm [25].

We have previously observed that in a long-term swimming assay [9], the average fraction of wild type animals in quiescence during the first phase (first 90 minutes) of swimming was low 0.02 +/- 0.004. In the subsequent 90 minutes the average fraction of worms in quiescence increased to 0.23 +/- 0.01. Loss-of-function mutants of either of calcineurin subunit *cnb-1(jh300) *or *tax-6(db60) *[25,26] exhibited a striking temporally changing phenotype in which a spike in fraction of animals in quiescence during the first 90 minutes (0.75 ± 0.2 for *cnb-1(jh103)*) were followed by a decrease in fraction of animals in quiescence (0.62 ± 0.05 for *cnb-1(jh103)*, p < 0.0001, Mann-Whitney U test) during the subsequent period of the assay. In other words a higher fraction of *cnb-1(jh103) *and *tax- 6(db60) *animals went into quiescence in the first 90 minutes of the assay and remained quiescent during the entire assay duration compared to wild type animals. Thus calcineurin activity is required for preventing animals from going into quiescence. Moreover, the increased quiescence duration in calcineurin loss-of-function mutants is indicative of a role of calcineurin in proper termination of quiescence. Interestingly, the decrease in the fraction of worms in quiescence exhibited by *cnb-1(jh103) *and *tax- 6(db60) *animals after approximately 90 minutes of the assay (Additional File 2) suggested distinct mechanisms underlying quiescence in the first and second phase of swimming.

**Figure 3:**
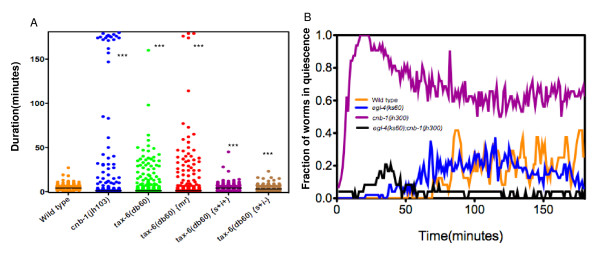
**Calcineurin activity in the sensory neurons is required for maintenance of swimming**. A) Distribution of quiescent bouts for Wild type (same as Figure 1A), *cnb-1(jh103)*, *tax-6(db60)*, *tax-6(db60)(mr)*, *tax-6(db60)(s+i) *and *tax-6(db60)(s+i-)*. *tax- 6(db60)(mr) *(n = 48 worms), *tax-6(db60)(s+i+) *(n = 35 worms) and *tax-6(db60)(s+i-) *(n = 37 worms) refers to a wild type *tax-6 *cDNA expressed in muscles (under *myo-3 *promoter), sensory and interneurons (under *unc-14p *promoter) and sensory neurons (under 1.1 Kb fragment of *tax-6 *promoter) respectively in a *tax-6(db60) *mutant background. Each point represents a single quiescent bout. Medians are shown as horizontal black bars (for means and errors see Table 1). *** indicates significantly different from wild type p < 0.0001 as determined by Kruskal-Wallis test follwed by Dunn's multiple comparison. B) PKG and calcineurin genetically interact to regulate swim quiescence pattern. Fraction of worms in quiescence versus time for wild type (same as Figure 1B), *egl- 4(ks60) *(same as Figure 1B), *cnb-1(jh103) *(n = 24), *egl-4(ks60);cnb-1(jh103) *(n = 24) worms.

In order to determine whether calcineurin activity was required in the nervous system or in other tissues to maintain swimming, we expressed a wild type *tax-6 *transgene under tissue specific promoters in *tax-6(db60)) *mutants. When expressed under the *myo-3 *promoter thereby restricting the expression of *tax-6 *to the body-wall muscles, the increased quiescence phenotype of *tax-6(db60) *mutants was not rescued (Figure 3A). However, when *tax-6 *cDNA was expressed in sensory neurons alone from a 1.1 kb fragment of the *tax-6 *promoter or both sensory and interneurons from an *unc-14 *promoter [27], the increased quiescence phenotype of *tax-6(db60) *was rescued, suggesting *tax-6 *activity in the sensory neurons is critical for maintenance of swimming and proper quiescence duration. Sensory neuronal activity of *tax-6 *was sufficient for generation of wild type quiescence bouts (Figure 3A, data not shown). It is possible that, in a *tax-6 *mutant, unopposed phosphorylation by a kinase promotes quiescence. Taken together, these data suggest that activity of calcineurin in the sensory neurons is necessary and sufficient for maintaining a wild type swimming-quiescence pattern in liquid. The altered behavioral patterns in the first and second phases of swimming in calcineurin mutants are likely due to defects in the sensory neurons.

### PKG and calcineurin genetically interact to regulate quiescence

To investigate whether the quiescence induced due to loss of calcineurin activity was dependent on *egl-4*, we assayed *cnb-1(jh103); egl-4(ks60) *double mutants. A significantly lower fraction of worms (0.05 ± 0.04) went into and remained in quiescence compared to the *cnb-1 *mutant worms (0.62 ± 0.05) throughout the assay suggesting that *egl-4 *acts genetically downstream of or parallel to *cnb-1 *in promoting the quiescent state (Figure 3B). Since calcineurin loss-of-function resulted in increased quiescent bout duration, which was dependent on PKG function, our data is consistent with a mechanism where events mediated by unopposed phosphorylation by *egl-4 *in the *cnb-1 *loss-of-function mutant increases quiescent bout durations.

## Discussion

We investigated the genetic basis of a striking behavioral pattern displayed by swimming nematodes in liquid in which they undergo alternate bouts of swimming and quiescence. We found that like other calcineurin-modulated behaviors in *C elegans*, activity of calcineurin in the sensory neurons is critical to maintain swimming in *C. elegans *in liquid. In analogy with the neuronal properties that change in a calcineurin mutant, namely defect in gain control [25], it is likely that increased quiescence in the *tax- 6/cnb-1 *loss-of-function mutants is due to hyperactivation of one or more sensory neurons. In addition, calcineurin mutants displayed a temporally changing phenotype in which there was a significant drop in the fraction of worms in quiescence after 90 minutes. This recovery after 90 minutes from start of assay was more pronounced in case of *tax-6(db60) *mutants than *cnb-1(jh300) *mutants, suggesting other phosphatases or autoregulatory mechanisms may play a role in shaping the temporal pattern of swim- quiescence pattern in liquid. It is possible that in *tax-6 *loss-of-function mutants unopposed phosphorylation of a yet unidentified substrate induces prolonged quiescence. The phosphorylation possibly involves *egl-4 *activity as the increased quiescence in calcineurin loss of function mutants was suppressed by loss of function muations in *egl-4*. During the first 90 minutes of the assay, PKG and calcineurin play opposing roles in maintenance of swimming. In the second phase (the subsequent 90 minutes) of the assay, however, the fraction of worms in quiescence in *egl-4;cnb-1 *double mutants was lower than the *egl-4 *loss of function mutants, suggesting that additional yet unidentified mechanisms may play a role in shaping the temporal pattern of quiescence during swimming in liquid.

We found that the protein kinase G homolog *egl-4 *acts in the sensory neurons to enhance the quiescence duration liquid. Similar to other paradigms of behavioral quiescence, we found that PKG activity in sensory neurons expressing the cGMP-gated calcium channel *tax-4 *is critical for regulating quiescent bout duration. However, beyond the involvement of *egl-4 *in *tax-4 *expressing neurons, the pathways for induction of different types of quiescence seem to be different. For instance, in quiescence induced by high quality food, loss-of-function mutants of *tax-2*, encoding the alpha subunit of the cGMP gated ion channels, exhibited very little quiescence [13]. However quiescence in liquid without food doesn't seem to involve participation of these channels as quiescence bouts of double mutants of alpha and beta subunits, *tax-2(p691);tax-4(ky89)*, were not different from wild type (Table 1), ruling out these channels as phosphorylation targets of *egl-4 *in modulating quiescence duration in liquid without food. This difference may be attributed to a different target of *egl-4 *in the sensory neurons or action of *egl-4 *in additional sites beyond the sensory neurons.

**Figure 4:**
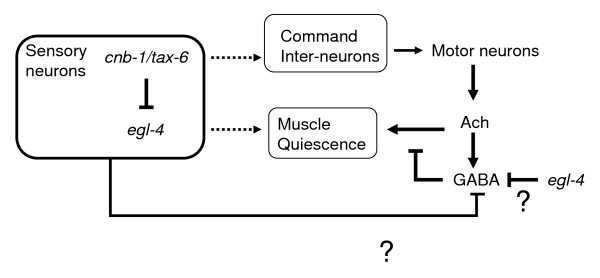
**Model for action of EGL-4 and TAX-6 in regulation of the quiescent state**. ACh = acetylcholine. Calcineurin (*tax-6*) facilitates maintenance of swimming by its activity in the sensory neurons. PKG (*egl-4*) and calcineurin (*tax-6) *acts antagonistically in the sensory neurons. In addition, *egl-4 *activity outside the sensory neurons antagonizes GABAergic activity that results in termination of quiescence. Alternatively *egl-4 *may activate a neurosecretory pathway in the sensory neurons that may block GABAergic neurotransmission.

Our data also suggests a second site of action of *egl-4 *other than the sensory neurons. It seems likely that *egl-4 *may be acting functionally downstream of acetylcholine release at an as yet unidentified site in regulating quiescence bouts. Our hypothesis is supported by the lack of complete rescue of *egl-4 *loss of function by expression in the sensory neurons, the suppression in *egl-4 *mutants of increased quiescence resulting from manipulations of the command interneurons as well as lack of suppression of the GABAergic loss of function mutants. These observations support a site of action of EGL-4 other than within sensory neurons. One possibility is that during quiescence, *egl-4 *inhibits GABA-ergic signaling and therefore signaling through the ACh pathway is increased causing quiescence. According to this model, in an *egl-4 *loss-of-function mutant GABA-ergic signaling is enhanced, which terminates quiescence prematurely (Figure 4). It is also possible that *egl-4 *acts solely in the sensory neurons by activating a neurosecretory pathway that inhibits GABAergic activity (Figure 4). This is consistent with two pathways in maintenance of quiescence. However this scenario requires that sensory neurons with global defects in cilia result in excessive activation of the neurosecretory pathway. Further studies are required to distinguish between these two models. The data is also consistent with a possibility that partial loss of function mutations in *egl-4 *counters "gain-of-function" like situation that may be mimicked by aldicarb or constitutive depolarization of command interneurons. However this is less likely as increased quiescence due to loss of function mutations in *nmr-1*, *osm-3 *and osm-6 was suppressed by *egl-4 *loss of function mutants.

## Conclusions

In summary, we have identified parts of a genetic network involving calcineurin and PKG that generate a temporal pattern during swimming in *C.elegans*. Though *egl-4 *has been implicated in all paradigms of behavioral quiescence in *C. elegans*, our results indicate that the target of EGL-4 in regulating quiescence in liquid is distinct from the other forms of quiescence. Based on our results we propose dual function of *egl-4*: one in the sensory neurons that is inhibited by calcineurin and another in inhibiting GABAergic neurotransmission (Figure 4). Our study also implicates opposite roles of a kinase and a phosphatase in generating temporal pattern in behavior. It is likely that the activity of kinase(s) increases the probability of the motor circuit to switch to a quiescent state. Such a process is opposed by calcineurin phosphatase activity in the sensory neurons. Studies of temporal patterns generated by intermittent motor activities have explored statistical properties of the behavioral state transitions or neuro-physiological properties of neural circuits underlying them [1,5,28]. However, very little is known about the genetics of the behavioral intermittencies over time. Our results in a genetically tractable organism establish a framework for further dissection of episodic behaviors.

## Methods

### Strains

Nematodes were reared and maintained at 20°C on *E. coli *OP50 on NGM agar plates according to published procedures [29]. Strains that were used are as follows: *tax- 6(db60), tax-6(db60) [myo3::tax-6], tax-6(db60) [tax-6sensory+inter+::tax-6], tax- 6(db60) [tax-6 sensory+inter-::tax-6] *were a kind gift from Ikue Mori (see Figure legend 3 for description of the promoters). The *egl-4 *transgenic rescue strains (*egl-4(n479) [odr- 3::egl-4], egl-4(n479) [odr-1::egl-4] *and *egl-4(n479) [tax-4::egl-4]*) were generously provided by David Raizen. *akIs9 *and *tax-2(p691);tax-4(ky89) *were kindly provided by A.V. Maricq and Cornelia Bargmann respectively. All other strains were either made during this work or obtained from CGC. Double mutants were generated using standard genetic procedures. *osm-6(p811) *and *osm-3(p802) *mutants were monitored by the inability of their sensory neurons to uptake the fluorescent dye DiO [24]. *cnb-1(jh103), egl-4(ks60) *and *nmr-(ak4) *deletions were confirmed by the following primer pairs:

Left oligo cnb-1: TCTTCTTGTGCACTTCGGTG

Right oligo cnb-1: CAACACAGCCGATCAAATG

EGL-4(KS60) FD: GAAACCTCCAATTCTGCCGAAGG

EGL-4(KS60) RV: GAATTTCCAGTCAACCAAATTCATAC

Nmr-1 (ak4) left: GGAAGAGTTTGAAAAACGGCG

Nmr-1 (ak4) right: CGTGTTCTTAGCTCACAGTGTCG

### Swimming assays

Swimming assays were performed essentially as described previously [9]. Briefly, one day old adults were transferred to a fresh unseeded plate at 25°C and allowed to forage for 2 min, after which they were picked singly into wells of a microtiter plate containing 200 μl M9 buffer at 25°C. The assays were carried out in a room where the temperature was maintained at 25 ± 1°C. Quiescence was defined as before [9]. Briefly less than two body bends per 5 seconds of observation were considered as quiescent irrespective of movement of the nose/head.

### Aldicarb and dye-filing assays

Aldicarb assays in liquid were performed as described before [9]. Dye filing assays were done essentially as published elsewhere with 1;2000 diluted solution of DiO (10 mg/ml stock) [24].

### Data analysis

Statistical analysis on the data was done using the Graphpad Prism version 5.0a software for Macintosh. For most cases the data was not normally distributed as determined by the by the D'Agastino and Pearson method. Unless otherwise mentioned we used the non parametric Mann Whitney U test for the quiescence bout durations for two genotypes or Kruskal-Wallis test followed by Dunn's multiple comparison for more than two genotypes. We also used the non-parametric Wilcoxon signed rank test to compare the medians of any given genotype against a hypothetical median of 4.500, which was not significantly different from the median for wild type worms.

## Authors' contributions

RG and SWE conceived the study, designed the experiments and wrote the manuscript. RG performed the experiments. Both authors read and approved the final manuscript.

## Supplementary Material

Additional file 1 Distribution of frequency of quiescent bout durations for *unc-25(sa94ts) and unc-25(sa94ts);egl-4(ks60) *worms with bin size = 3 minutes. Click here for file

Additional file 2 Fraction of worms in quiescence versus time for wild type (n = 25), *cnb- 1(jh103) *(n = 24), *tax-6(db60) *(n = 24) worms. Click here for file
