# Supplementary material for: Calcineurin and Protein kinase G regulate C. elegans behavioral quiescence during locomotion in liquid
Source: BMC Genet. 2010 Jan 27;11:7. doi: 10.1186/1471-2156-11-7 (PMC2834598; doi:10.1186/1471-2156-11-7)
Supplement: Additional file 1 — Distribution of frequency of quiescent bout durations for unc-25(sa94ts) and unc-25(sa94ts);egl-4(ks60) worms with bin size = 3 minutes. [file 1471-2156-11-7-S1.PDF]

Additional file 1

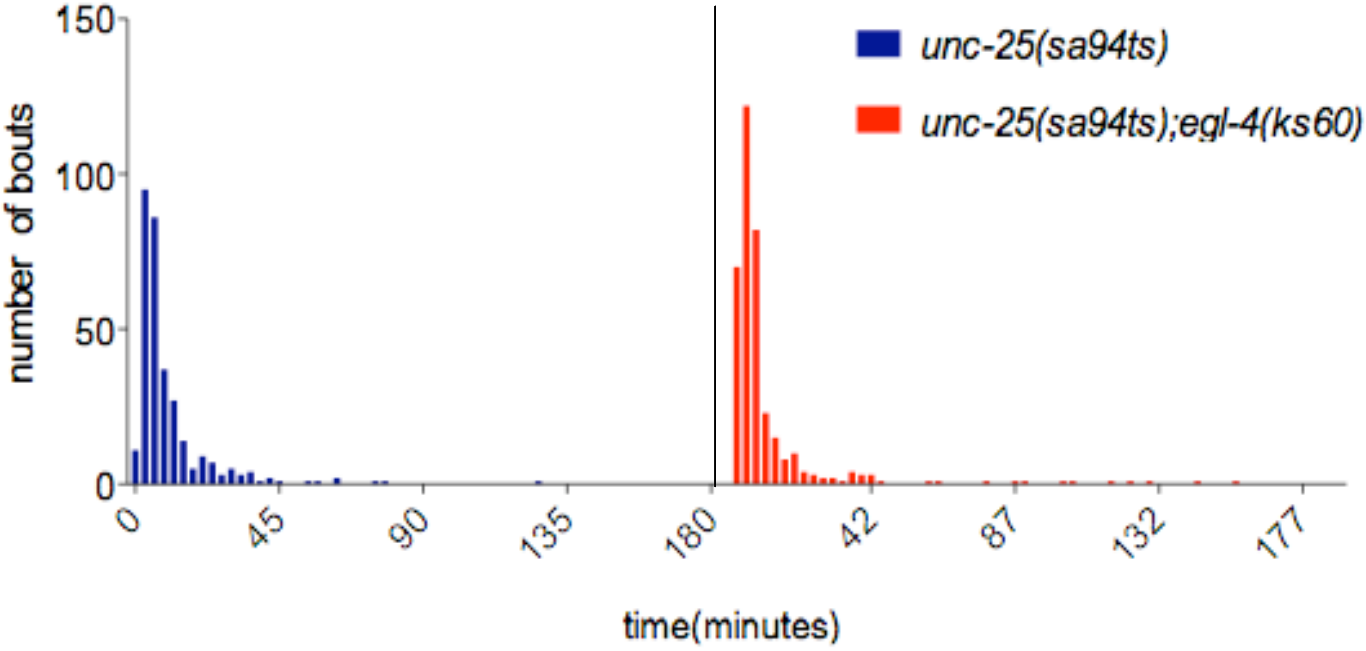

Distribution of frequency of quiescent bout durations for *unc-25(sa94ts) and unc-25(sa94ts);egl-4(ks60)* worms with bin size =3 minutes.
