# Supplementary material for: Calcineurin and Protein kinase G regulate C. elegans behavioral quiescence during locomotion in liquid
Source: BMC Genet. 2010 Jan 27;11:7. doi: 10.1186/1471-2156-11-7 (PMC2834598; doi:10.1186/1471-2156-11-7)
Supplement: Additional file 2 — Fraction of worms in quiescence versus time for wild type (n = 25), cnb- 1(jh103) (n = 24), tax-6(db60) (n = 24) worms. [file 1471-2156-11-7-S2.PDF]

Additional file 2

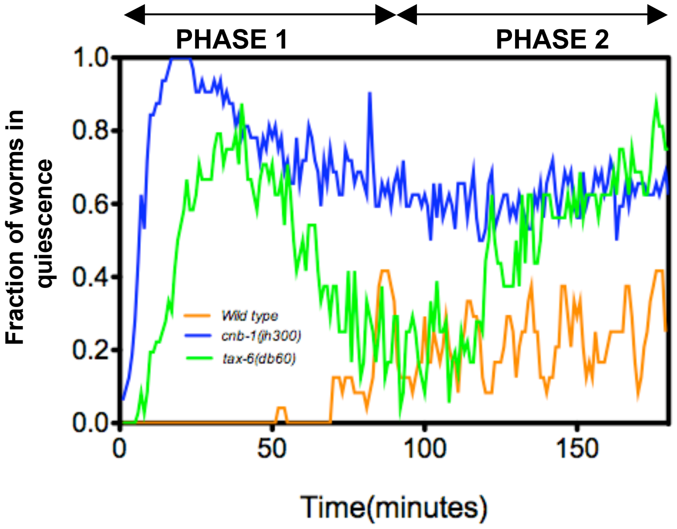

Fraction of worms in quiescence versus time for wild-type (n=25), *cnb-1(jh103)* (n=24), *tax-6(db60)* (n=24) worms.
